# Effects of stress on neural processing of combat-related stimuli in deployed soldiers: an fMRI study

**DOI:** 10.1038/s41398-022-02241-0

**Published:** 2022-11-17

**Authors:** Robert C. Lorenz, Oisin Butler, Gerd Willmund, Ulrich Wesemann, Peter Zimmermann, Jürgen Gallinat, Simone Kühn

**Affiliations:** 1 Lise Meitner Group for Environmental Neuroscience, Max Planck Institute for Human Development, Lentzeallee 94, 14195 Berlin, Germany; 2 Max Planck Dahlem Campus of Cognition (MPDCC), Dillenburgerstr. 53, 14199 Berlin, Germany; 3 Center for Military Mental Health, Military Hospital Berlin, Scharnhorststr. 13, 10115 Berlin, Germany; 4 University Medical Center Hamburg-Eppendorf (UKE), Department of Psychiatry and Psychotherapy W37, Martinistrasse 52, 20246 Hamburg, Germany; 5 Max Planck-UCL Center for Computational Psychiatry and Ageing Research, Lentzeallee 94, 14195 Berlin, Germany

**Keywords:** Learning and memory, Depression

## Abstract

Severe trauma exposure may lead to symptoms of both posttraumatic stress disorder and depression. Neuroanatomical theories suggest that both disorders may share imbalances in fronto-limbic circuits. Longitudinal studies are necessary to better understand the impact of a stressful life situation on potential long-term fronto-limbic imbalances. Here we investigated soldiers neural processing of combat-related stimuli versus negative affective stimuli before and after the deployment in different war zones. In the final analysis we included 104 deployed soldiers (combat group) and 36 soldiers that were not deployed (control group). Behaviorally, we found a significant group by time interaction regarding depression symptom scores with an increase in the combat group. Depressive symptoms were subclinical. On the neural level, neither the whole brain analysis nor the region of interest (ROI) analyses including frontal and limbic ROIs revealed any significant results in the group by time interaction. However, extracted ROI values of the group by time interaction of amygdala and hippocampus were positively associated with the change in depression symptom scores in the combat group, but not in the control group. These results highlight the role of depression in individuals that experience stressful life situations. Future studies may need to investigate the role of depressive symptoms after trauma exposure with different tasks that may be particularly sensitive to changes due to depressive symptoms.

## Introduction

The involvement in combat is an extremely stressful life experience and may have negative impact on physical and mental health. Soldiers are repeatedly faced with such stressful life situations during deployment. Individuals can differently cope with such extreme life experiences: while some are resilient [1], some develop symptoms of posttraumatic stress disorder (PTSD) and some even develop depressive symptoms [2]. Indeed, there is a high comorbidity (>50%) of depression among PTSD patients [3] and major depression (MDD) and PTSD symptoms are also frequently reported in military forces [4, 5].

Besides the high comorbidity of MDD and PTSD, both mental illnesses share common mechanisms, in particular an abnormal processing of affective information. In MDD and PTSD, a heightened reactivity to negative stimuli is observed as well as impairments in the voluntary regulation of emotions [6]. Considering the underlying neural processes of these affective disturbances, in MDD theoretical models conceptualizing a limbic-frontal imbalance focus on the amygdala, anterior cingulate cortex (ACC), and prefrontal cortex (PFC) [6, 7]. While some meta-analyses [8, 9] showed hyperreactivity in the amygdala, a recent comprehensive meta-analysis [10] highlighted the heterogeneity in MDD neuroimaging studies and the investigated heterogeneous clinical populations. In PTSD research, a prominent neuroanatomical theory suggests a hyperresponsivity in amygdala to fear-related stimuli that is accompanied by an inadequate prefrontal emotion regulation (associated with ventromedial PFC (VMPFC), ACC, medial PFC, subcallosal cortex and orbitofrontal cortex (OFC)) [11–13].

Considering neuroimaging studies with soldiers that were deployed to regions of combat and therefore trauma-exposed showed aberrant neural processing in particular in limbic-frontal areas. Different tasks with affective stimuli were used to investigate emotion processing: Van Wingen and colleagues [14] used a cognitive task with negative emotional stimuli (versus control stimuli), van Rooij et al. [15] presented positive, negative and neutral affective pictures, whereas White et al. [16] used an affective version of the Stroop task. The brain areas that were sensitive to deployment (exposure to trauma) in these studies were predominantly amygdala, insula and ACC. Furthermore, the military staff that were investigated in these studies was combat-exposed, but did not develop PTSD (in van Rooij et al. [15] groups were separated in veterans with and without PTSD).

The repeated experience of stress during deployment may lead to the above mentioned affective disturbances. Symptom provocation is a well-investigated method to study the changes in processing of trauma-related stimuli in PTSD research [12]. However, combat-related stimuli itself are not neutral, because they explicate a form of violence. Therefore, negative affective stimuli should be used in order to control for the common natural negative association between combat-stimuli and negative affect. The aim of the study was to investigate the specific change that is related to the prolonged stress due to deployment, which is reflected in processing of combat-stimuli, while controlling for the more general negative affective processing. We hypothesized that these changes may be related to limbic-frontal imbalances, even in individuals that do not develop MDD or PTSD. Taken together, in the current study we investigated, whether any kind of aberrant affective processing is present during the processing of combat-related stimuli (when controlling for negative affect) in a sample of soldiers that were deployed versus a non-deployed military control group.

## Methods

### Participants

The participants were consecutively recruited by the German military that contacted military units scheduled for deployment. Before the start of the study, the study was described by the recruitment team in detail and written informed consent was obtained from all participants that were willing to participate and were eligible. Eligibility criteria were no medical history of psychiatric, neurological or any severe medical condition and standard MRI eligibility criteria (e.g. no ferromagnetic implant).

Study visits, including fMRI assessment, were planned before and after deployment. We were able to collect data from 121 soldiers. Due to technical failure during data collection four participants had to be excluded. During data analysis another 13 participants had to be excluded due to excessive head movement during fMRI scanning (>3.5 mm translation or more than 3° rotation). The resulting combat group comprised 104 soldiers that were deployed to Afghanistan, Iraq, Kosovo or Mali between 2012 and 2017 for on average 131 days (SD = 39.8 days; about 4.3 months). The mean interval between the study visits before and after deployment was 213 days (SD = 67.9 days).

We also collected data from a military control group. After excluding five participants of the control group due to head movement issues (same cutoff values as in the combat group) 36 participants remained. The participants in the control group were also soldiers, which were scanned twice as well with a mean time window of 226 days (SD = 112.9 days) between the study visits without any deployment in that period.

The sample size calculation was based on a repeated-measures ANOVA (two time points, two groups) assuming an effect size of *f* = 0.3, and a power of 0.95. In order to enable potential subgroup analyses in the deployed group, the sample size was chosen to be larger than that of the control group.

The study was conducted in accordance with the declaration of Helsinki and approved by the local ethics committee of Charité University Clinic.

Baseline symptom scores were assessed for PTSD and MDD. The Post-traumatic Diagnostic Scale was used (PDS [17]) for PTSD assessment. All participants scored below the cut-off for a suggested clinical diagnosis (cut-off = 28 [18], and also below a more conservative cut-off value of 24 as suggested for a German sample [19]). For MDD, Beck Depression Inventory II (BDI-II [20]) was applied. Again, all participants scored below a cutoff (18 was used, because of best diagnostic properties regarding sensitivity and specificity [21]). All demographic variables can be found in Table 1.

**Table 1:** Characteristics of the investigated sample at baseline (upper rows), change in clinical questionnaires across groups and time, rating of the experimental stimuli across groups and time.

	Stress group (*n* = 104)		Control group (*n* = 36)		Statistics	
	Baseline	Follow-up	Baseline	Follow-up	Baseline	Group x time^1^
	Mean (SD)	Mean (SD)	Mean (SD)	Mean (SD)	test- and *p*-value	*F*- and *p*-value
**Baseline characteristics**						
Age (years)	32.7 (8.3)		29.6 (6.3)		*T* = 2.07; *p* = 0.04*	
Sex (m/f)	97/7		32/4		*χ*^*2*^ = 0.71; *p* = 0.473	
Lifetime days of deployment	195.1 (293.2)		123 (202.2)		*T* = 1.37; *p* = 0.174	
No. of subjects with previous deployments (yes/no)	59/45		16/20		*χ*^*2*^ = 1.62; *p* = 0.246	
Digit symbol score	54.3 (11.4)		55.6 (12.7)		*T* = −0.58; *p* = 0.563	
**Psychological questionnaires**						
PTSD symptomatology (PDS Total)	2.49 (3.4)	2.33 (3.6)	3.28 (4.8)	2.29 (3.9)	*T* = −0.949; *p* = 0.345	*F* = 1.31; *p* = 0.255
Anxiety (STAI State)	31.6 (6.3)	32.3 (7.4)	34.1 (5.9)	33.8 (6.5)	*T* = −2.034; *p* = 0.044*	*F* = 0.38; *p* = 0.54
Anxiety Sensitivity (ASI)	14.4 (7.6)	13.7 (7.7)	13.6 (7.7)	12.9 (6.8)	*T* = 0.54; *p* = 0.593	*F* = 0.07; *p* = 0.797
Depressive symptoms (BDI-II)	2.54 (3.1)	3.24 (4.1)	3.06 (3.8)	2.47 (3.9)	*T* = −0.82; *p* = 0.413	*F* = 5.45; *p* = 0.021*
Rumination (RSQ)	33.6 (7.6)	33.3 (8.8)	33.1 (9.4)	33.6 (8.8)	*T* = 0.32; *p* = 0.748	*F* = 0.75; *p* = 0.387
**Rating of experimental stimuli**						
Combat arousal	1.95 (0.55)	1.83 (0.56)	2.08 (0.59)	1.92 (0.63)	*T* = −1.195; *p* = 0.234	*F* = 0.1; *p* = 0.756
Combat valence	1.61 (0.45)	1.45 (0.4)	1.68 (0.44)	1.55 (0.43)	*T* = −0.773; *p* = 0.441	*F* = 0.12; *p* = 0.732
IAPS arousal	2.13 (0.44)	1.95 (0.5)	2.1 (0.47)	1.95 (0.53)	*T* = 0.369; *p* = 0.713	*F* = 0.25; *p* = 0.616
IAPS valence	2.2 (0.43)	2.1 (0.48)	2.18 (0.49)	2.14 (0.55)	*T* = 0.205; *p* = 0.838	*F* = 0.62; *p* = 0.432

^1^Group x time resulting from a repeated measures analysis of variance; for psychological questionnaires age and STAI state at baseline were used as covariate (except for STAT state itself; here only age was used as covariate).

### Questionnaires

In order to characterize the sample we applied different self-report questionnaires. These were comprised of Anxiety Sensitivity Index (ASI), BDI-II, Combat Experience Scale (CES), Response Styles Questionnaire (RSQ), PDS, and State Trait Anxiety Inventory state (STAI).

PDS [17] was used to assess PTSD symptom severity. The self-report questionnaire includes 17 items that target main symptoms of PTSD experienced in the past 30 days using a 4-point scale ranging from 0 (“not at all or only one time”) to 3 (“5 or more times a week/almost always”).

BDI-II [20] was designed to assess to behaviors and symptoms of depression experienced in the past 2 weeks. Participants were asked to rate each of the 21 items on a four-point scale. Higher BDI-II scores indicate more severe depressive symptoms.

Description of the other mentioned questionnaires can be found in the supplementary material.

### Experimental Task

**Fig. 1:**
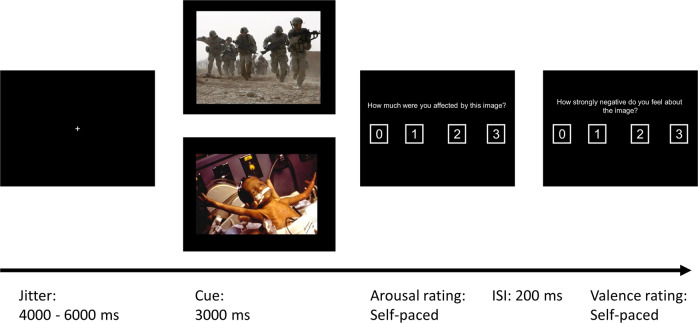
Task design. After a variable jitter combat or negative affective IAPS stimuli were presented for 3 s at the beginning of each trial. Arousal and valence rating (order randomized) each with a 4-point-scale were followed with a 200 ms interval (fixation cross, not shown) between the ratings.

In order to assess the neural signature of combat-related versus negative affective stimulus processing, we used a typical event-related cue reactivity task including a valence and arousal rating (see Fig. 1).

#### Image stimuli

For the trauma-related stimuli, we used thirty images of genuine war photographs provided by the German Armed Forces. Images were taken during active duty (mainly in Afghanistan) by soldiers. These stimuli were used beforehand in an fMRI study involving cognitive reappraisal and expressive suppression and showing robust neural responses in the limbic-frontal target areas [22]. This stimulus set included pictures of military vehicles, explosions, soldiers on standby to fire and military emergency care units. Photos of explicit presentations of death or injury were excluded to avoid a potential trigger of flashbacks.

For negative affective stimuli, thirty pictures from the International Affective Picture System (IAPS) [23] with unpleasant valence (according to IAPS manual: *M* = 2.91, SD = 0.66; min = 1.79; max = 4.07) and different arousal levels (15 images with lower arousal levels, i.e. <4.5 and 15 images with higher arousal levels, i.e. >5) were selected (according to IAPS manual: *M* = 4.86, SD = 0.94; min = 2.63; max = 6.61). Examples included photos of sick people, sad people, destroyed houses (earth quake), jail, and environmental pollution. For both stimulus sets, six example pictures are shown in the Supplementary Fig. S1. IAPS picture numbers are listed in the supplement.

#### Task description

Each trial started with a presentation of a fixation cross. The length of the fixation was randomly jittered between 4 and 6 s. Consequently, depending on the condition a combat-related or negative affect image was presented for 3 s. The sequence of image condition was counterbalanced in a pseudo-random manner across participants and measurement time points. Immediately after the image presentation, participants were asked to rate the arousal on a four-point scale “How much were you affected by this image?” (German: Wie sehr hat Sie dieses Bild bewegt?), with 1 indicating “not affected” and 4 indicating “strongly affected”. Participants had to respond with their right hand on a four button response box and were instructed to respond as fast and spontaneously as possible. Right after the participants‘ response a fixation cross was presented for 200 ms followed by the valence rating question. Participants were asked in the second question “How strongly negative do you feel about the image?” (German: Wie stark negativ empfinden Sie das Bild?), again participants responded on a four-point scale with 1 indicating “not strong at all” and 4 indicating “very strong”. The order of the rating questions (arousal-valence or valence-arousal) as well as the visually presented order of the four response options (1-2-3-4 or 4-3-2-1) was presented randomly in order to avoid movement preparation. After the two self-paced rating questions, the next trial started beginning with a fixation cross. Sixty trials were presented in the experimental task. Due to the self-paced nature of the rating, the task length varied individually with a mean length of 12.52 min (SD = 80.9 s).

### Imaging procedure

Magnetic resonance images were acquired using a Siemens Tim Trio 3 T scanner and a standard 12-channel head coil. At the beginning of each scanning session, structural images (for normalization procedure) were obtained using a magnetization prepared gradient-echo sequence (MPRAGE) with these parameters: repetition time = 2500 ms; echo time = 4.77 ms; TI = 1100 ms, acquisition matrix = 256 × 256 × 176, flip angle = 7˚; 1 × 1 × 1 mm voxel size. For functional imaging, T2*-weighted echo-planar images (EPI) were collected (repetition time = 2000 ms, echo time = 30 ms, image matrix = 72 × 72, FOV = 216 mm, flip angle = 80°, slice thickness = 3 mm, slice order = interleaved, voxel size 3 x 3 x 3 mm voxel size, 36 axial slices). Experimental stimulation was presented via a video projector on a mirror system on top of the head coil and the software Presentation® (http://www.neurobs.com).

### Statistical analysis of imaging data

#### Image preprocessing

All images were converted from DICOM into Nifti format. Image preprocessing was conducted using Statistical Parametric Mapping 12 (SPM12; Wellcome Trust Center for Neuroimaging, London) based on MATLAB (Mathworks). The first five EPI images in each session were discarded to allow longitudinal magnetization to reach equilibrium. EPI images were slice-time and movement corrected as well as normalized into the stereotactic normalized standard space of the Montreal Neuroimaging Institute using the segmentation algorithm as implemented in SPM12. Finally, EPI images were spatially smoothed with a 3D Gaussian kernel of 6 mm full width at half maximum.

#### Statistical analysis

A two-stage mixed-effects general linear model (GLM) was implemented. For each participant and time point (pre and post assessment) the model was comprised of separate independent variables for the two conditions (combat vs. negative affect) with a fixed length of 3 s, two separate independent variables for the arousal and valence rating, an independent variable for button presses, and six independent variables for the rigid body movement parameters. The target of the group analysis was two-fold. First, we wanted to show the neural signature of processing combat and negative affect images in general (task effect) and, therefore, for each participant we calculated a baseline contrast image for each of the two conditions for the pre assessment. On a group level, *t*-tests were conducted. Second, we wanted to show the interaction group by time and, therefore, calculated differential contrast images for the combat condition versus the negative affect condition for each time point. These differential T-contrast images were entered on group level analysis into a flexible factorial analysis of variance with the factors group (deployment versus control) and time (pre and post assessment). Whole brain group effects were reported with a statistical threshold of *p* < 0.05 using family-wise error correction (FWE).

#### Region of interest analyses

As outlined in the introduction, we had an a priori hypothesis of limbic-frontal imbalances in the current study. In particular, we were interested in the limbic regions amygdala and hippocampus and in the frontal regions ACC, subcallosal ACC, VMPFC and inferior frontal gyrus (extending in anterior insular cortex). For the limbic regions, we used the anatomical regions of interest (ROI) as described in the Anatomic Labeling brain atlas [24]. However, anatomical regions in the prefrontal regions are often very broad and the borders are varying depending on the neuroanatomical atlas. Therefore, we aimed to find functional literature imaging ROIs of the prefrontal regions. We extracted the meta-analysis for the term “negative affect” from the website neurosynth (an automated syntheses tool for fMRI data [25], www.neurosynth.org) that synthesized data from 97 studies about processing of negative affective information (uniformity test). From this whole brain meta-analysis we extracted the ROIs for the above mentioned regions. With this approach we ensured to extract only these parts of the cortical areas of interest, that were previously shown to be involved in processing of negative affective information. Additionally, we conducted a subgroup analyses with the extracted ROI data and separated the combat group in four groups according to different experiences of combat (see supplementary material).

### Statistical analyses of behavioral data

For the self-report questionnaires, we conducted an ANCOVA for repeated measurements with the factors group (between-subject factor) and time (within-subject factor) while controlling for age and state anxiety at pretest (STAI state), since we observed a significant group difference in these variables at pretest. For the behavioral rating data of the experimental task (arousal and valence rating) we also conducted a repeated-measure ANCOVA with the same factors.

Furthermore, for the change of symptoms of PTSD and MDD, we calculated change scores (posttest versus pretest) for the questionnaires PDS and BDI-II. In order to test, whether these changes in symptoms of PTSD or MDD may be related to limbic-frontal imbalances, we correlated the change scores of the questionnaires with change scores that were extracted from the ROIs of the fMRI analysis by using the contrast posttest[combat > IAPS] > pretest[combat > IAPS] for each participant. We report the results according to a significance threshold of *p* < 0.05 (uncorrected) as well as a significance threshold of *p* < 0.05 corrected for multiple comparisons (Bonferroni correction for 9 ROIs). IBM® SPSS® Statistics Version 25 was used for all behavioral data analyses.

## Results

### Behavioral results

Changes in the Combat Experience Scale were screened before and after deployment. Military service members most frequently reported the following after deployment: saw destroyed homes and villages, hostile reactions from civilians, saw dead bodies or human remains, saw ill/injured women or children whom the soldiers were not able to help, and soldiers received incoming artillery, rocket, or mortar fire.

Baseline characteristics and behavioral results are shown in Table 1. According to the baseline characteristics, combat and control group did not differ from each other except for age (*t* = 2.07; *p* = 0.04) and STAI state (*t* = −2.034; *p* = 0.044). Consequently, we used age and STAI state (pretest) as covariate in the group by time ANOVAs for the psychological questionnaires (except for STAT state itself; here only age was used as a covariate). For PTSD and MDD symptom scores, we found no interaction effect for PTSD scores (PDS Total), but a significant interaction for MDD scores (BDI-II), with an increase (*t*(103) = −2.31, *p* = 0.023) in the combat group and a descriptive, non-significant decrease in the control group (*t*(35) = 1.16, *p* = 0.255). At posttest all participants scored below the cut-off in the PDS (cutoff 24) and below in the BDI-II (cutoff 18) except one participant (BDI-II = 19, combat group). In the other questionnaires, namely state anxiety (STAI State), anxiety sensitivity (ASI), and rumination (RSQ), we found no significant results.

Considering the rating of the stimuli during the fMRI experiment, we found neither significant group by time interactions for combat stimuli (for both arousal and valence rating), nor for IAPS stimuli (both ratings).

### Imaging results

The results for the fMRI analyses are presented in three steps. First, processing of combat and negative affect images in general is presented (task effect). Second, whole brain and ROI analyses for group by time interaction are presented. Finally, the extracted parameter estimates of the ROI analysis (group by time interaction) were correlated with behavioral data, in particular with PTSD and MDD symptom scores (PDS and BDI-II).

#### Task effect

At baseline and across all participants during processing of combat and IAPS stimuli brain activity in a largely overlapping network was observed in the following areas: visual cortex extending into visual and dorsal stream, precuneus, parietal areas (superior parietal cortex, inferior parietal cortex), temporal areas (middle and superior temporal gyrus), frontal areas (supplementary motor cortex, cingulate cortex, medial PFC, VMPFC, IFG extending into insular cortex, precentral gyrus, and superior frontal gyrus), and subcortical areas (thalamus, caudate, amygdala, and hippocampus). When contrasting the processing of both image types, we found stronger activity in precuneus (extending into post cingulate cortex, calcarine gyrus, fusiform gyrus and parahippocampal gyrus), middle frontal gyrus, and VMPFC in combat compared to IAPS stimuli. Stronger activity related to IAPS as compared to combat stimuli was observed in visual cortex, supramarginal gyrus, ACC extending into supplementary motor cortex and medial PFC, IFG extending into insular cortex and subcortical areas (caudate, amygdala). See Fig. S2 and Tables S1-S4 in the Supplementary material.

#### Posttest and group by time interaction

**Fig. 2:**
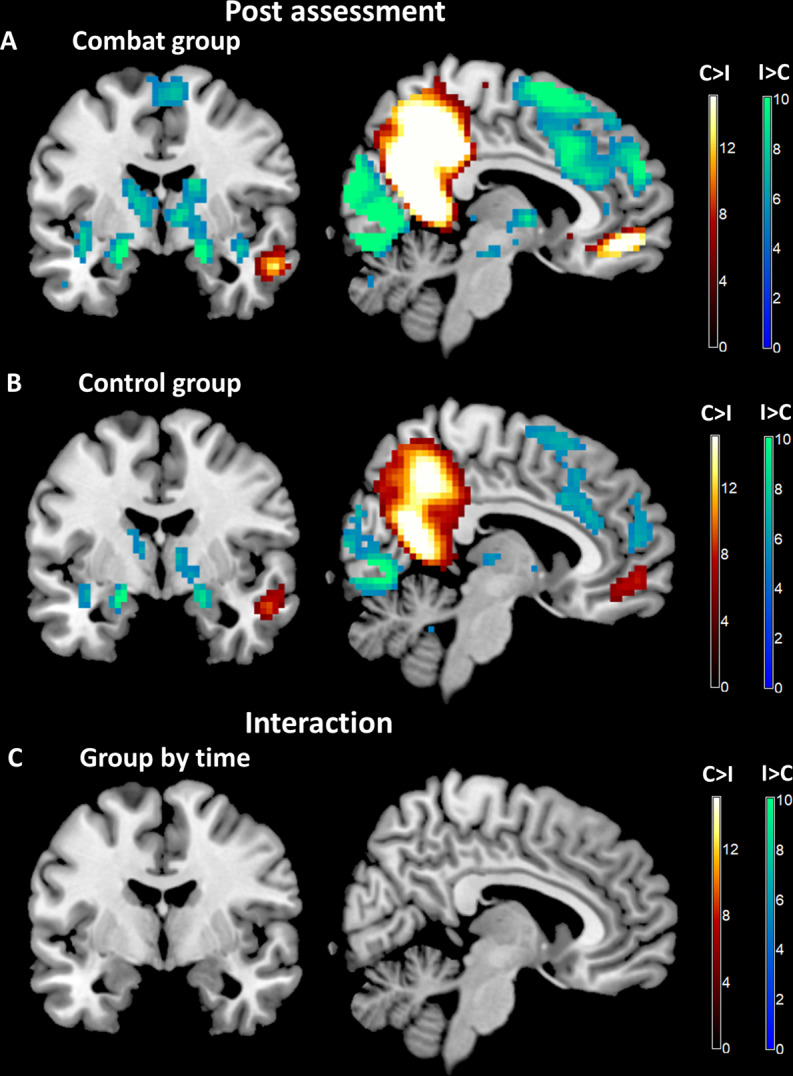
Imaging results, posttest for each group and group by time interaction. **A**, **B** Very similar differential effects between processing of combat versus negative affective IAPS stimuli were observed in the combat and control group. **C** Group (combat vs. control group) by time (pretest versus posttest) interaction revealed no significant differences.

Within each group (combat and control group) the activation pattern at posttest between the processing of combat and IAPS stimuli was comparable and also similar to the above reported contrast combat vs. IAPS stimuli at baseline across groups. The difference between the processing of the two stimulus types was more pronounced in the combat group (e.g. more widespread cluster). However, there were no significant voxels representing the group by time interaction in the whole brain analysis. See Fig. 2 and Tables S5-S8 in the Supplementary material.

#### ROI analyses

**Fig. 3:**
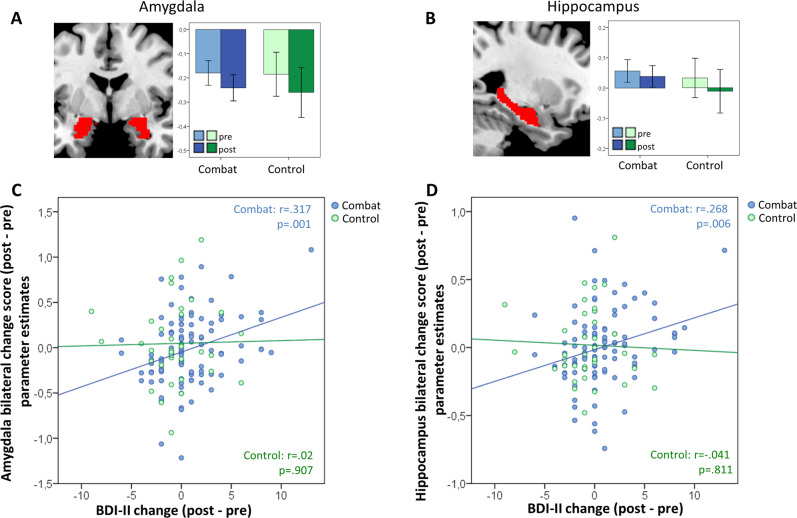
Imaging results, region of interest (ROI) analysis, and correlational analyses. **A**, **B** Anatomical location and bar graphs for the contrast combat versus negative affective IAPS stimuli for the two groups and time points were presented for the two a priori defined ROIs amygdala and hippocampus. In both regions no significant group (combat vs. control group) by time (pretest versus posttest) interactions were observed. Error bars in the bar graphs represent 95% confidence intervals. Results of other ROIs are shown in the supplementary material. **C**, **D** Associations of the change score of the ROI analyses and the behavioral analyses. On the *x*-axis the change of MDD symptom scores (posttest versus pretest) and on the *y*-axis the results from the ROIs of the (**C**) bilateral amygdala and the (**D**) bilateral hippocampus of the fMRI analysis using the interaction contrast posttest[combat > IAPS] > pretest[combat > IAPS] are shown. In both ROIs a significant correlation was observed in the combat group (shown in blue), but not in the control group (shown in green). For display purposes results in the figure are presented for bilateral amygdala and bilateral hippocampus. ROI analyses were calculated for right and left amygdala as well as right and left hippocampus separately (see text and Table 2).

According to our a priori hypotheses, we defined six regions of interest. Neither in frontal areas (ACC, subcallosal ACC, VMPFC, and IFG/insula) nor in subcortical areas (amygdala and hippocampus), a significant group by time by stimulus type interaction was observed (all *p*-values > 0.05). See Fig. 3, Supplementary Fig. S3, and supplementary table S9. We also did not find an effect of combat experience in the subgroup analyses of the ROI data (see Supplementary Material).

### Behavioral and imaging associations

Finally, we correlated the extracted mean ROI values for the change score for each participant and correlated these values with the change scores for the symptoms of MDD and PTSD. Significant correlations were exclusively found in subcortical areas in the combat group, in particular in the amygdala (Bonferroni corrected) and the hippocampus (Bonferroni corrected for right Hippocampus); see Table 2. The higher the increase in amygdala and hippocampal neural activity related to processing of combat-related versus negative affect-related pictures at posttest compared to pretest, the higher the increase in depressive symptoms. In contrast, no significant correlations were found in frontal areas and correlations in the control group were also not significant (all *p*-values > 0.05). See Fig. 3. Additionally, we tested for significant group differences in correlations in amygdala and hippocampus using one-sided *Z*-tests (according to https://www.psychometrica.de/correlation.html#independent) and found significant differences between the correlations of the two groups in left amygdala (*Z* = 2.243; *p* = 0.012) and right hippocampus (*Z* = 1.697; *p* = 0.045), while the difference in the left hippocampus was only almost significant (*Z* = 1.525; *p* = 0.064) and in right amygdala not significant (*Z* = 1.172; *p* = 0.121).

**Table 2:** Correlational analysis between imaging parameter estimates from the interaction group x time per anatomical region (sorted by frontal and subcortical regions) and the self-report questionnaires BDI-II and PDS for each group.

	Combat group		Control group	
Anatomical region	BDI-II (*n* = 104) *r*; *p*	PDS (*n* = 74) *r*; *p*	BDI-II (*n* = 36) *r*; *p*	PDS (*n* = 25) *r*; *p*
**Frontal regions**				
ACC	0.039; 0.695	0.187; 0.111	0.322; 0.055	−0.253; 0.221
Subcallosal ACC	0.163; 0.097	0.057; 0.628	0.115; 0.505	−0.074; 0.724
VMPFC	0.175; 0.075	0.106; 0.370	−0.125; 0.467	0.051; 0.808
Inferior frontal cortex / insula left	0.141; 0.153	0.074; 0.529	0.121; 0.482	−0.319; 0.120
Inferior frontal cortex / insula right	0.066; 0.504	0.033; 0.783	0.195; 0.254	−0.139; 0.508
**Subcortical regions**				
Amygdala left	0.316; 0.001**	−0.111; 0.345	−0.122; 0.478	−0.212; 0.309
Amygdala right	0.336; <0.001**	−0.074; 0.528	0.114; 0.508	0.016; 939
Hippocampus left	0.268; 0.006*	−0.025; 0.835	−0.031; 0.856	0.035; 0.868
Hippocampus right	0.287; 0.003**	−0.087; 0.461	−0.045; 0.793	−0.119; 0.572

*Significant according to significant threshold of *p* < 0.05 (uncorrected for multiple comparisons)

**Significant according to significant threshold of *p* < 0.05 (Bonferroni corrected for nine ROI)

## Discussion

In the current study we investigated the influence of a stressful life situation, in particular deployment of soldiers to different war zones, on the processing of combat-related stimuli versus negative affective stimuli in the brain. Behaviorally, we found a significant group by time interaction regarding depression symptom scores with an increase in the combat group. On the neural level, neither the whole brain analysis nor the ROI analyses revealed any significant results in the group by time interaction. However, change scores of extracted ROI values of the amygdala and hippocampus were positively associated with the change in depression scores in the combat group, but not in the control group. This shows that increases in amygdala and hippocampal functional reactivity during combat picture exposure compared with regular negative affect-related pictures were positively related to increases in symptoms of depression.

### MDD and PTSD symptom scores

Many soldiers of the combat group experienced combat-related stressors, however, an increase in PTSD symptom scores (in PDS or in other related questionnaires such as RSQ rumination or STAI state) was not observed. However, in the combat group MDD symptom scores were increased after deployment. Of note, the BDI-II scores were altogether relatively low, even at posttest in the combat group, even lower as compared to a non-clinical population [26]. Only one participant of the combat group scored above a cutoff value, thus the group average increase is not clinically relevant. The low scores may also reflect some kind of social desirability or even the fear of the label ‘mentally ill’ (stigma) [27].

The results in MDD and PTSD symptom scores of this study are in line with a previous longitudinal study showing no increase in PTSD, but in depressive symptoms over the course of deployment [28]. Also in the longitudinal study by van Wingen et al. [14], soldiers did not report increased PTSD scores after combat deployment. Considering the clinical diagnosis by applying a structured clinical interview, the 12-month PTSD prevalence of soldiers from German military was relatively low in two different groups, namely in deployed soldiers (2.9%) and in soldiers not deployed (1.2%) [29]. Thus, the observed low rates of clinical relevant PTSD and MDD in the current sample were also seen in previous studies.

### No implication on neurobiological mechanisms of combat-related PTSD

The military service members that were investigated in the current study did not show any clinically relevant symptoms of PTSD. However, it has been repeatedly shown that PTSD is increased in war veterans. Therefore, it is not valid to draw any conclusions from the current study to neurobiological mechanisms of combat-related PTSD.

### Rating of combat-related vs. negative affective stimuli

The arousal and valence ratings of both stimulus types used in the current study were not selectively altered by deployment as we found no group by time interaction. Noteworthy, the valence of the negative affective IAPS stimuli was rated slightly more negative than the combat stimuli by both groups. Thus, the combat stimuli did not trigger such strong negative emotions (as negative IAPS pictures) in the current sample. This also reflected neurally, because e.g. the amygdala was more strongly activated in IAPS stimuli compared to combat stimuli across all time points und groups. The results show that using negative affective stimuli possibly over-controls for the negative affect that combat-related stimuli have independently of a deployment.

### Neuroimaging findings: task effect

The contrast of combat versus negative affective stimuli revealed not only a stronger response to negative affective stimuli in limbic regions (i.e. in amygdala), but also in prefrontal areas, namely ACC extending into SMA and medial PFC as well as IFG extending into insula. These areas have in the literature been repeatedly described as a ventral attention network and are also involved in negative emotional processes (see e.g. a review by Lindquist et al. [30]).

On the other hand combat stimuli provoked a stronger neural response in posterior midline areas such as posterior cingulate cortex and precuneus as well as VMPFC. These structures are often reported in association with self-referencing [31, 32]. Indeed, the combat-related stimuli were highly self-relevant for both combat and control group, because participants of the control group were also soldiers and 16 (of 36) had been on a foreign deployment before the start of the study. This may be an explanation for the strong activation pattern in the self-reference network at posttest in both groups.

### Group by time interaction

In contrast to our hypothesis about limbic-frontal imbalances due to stressful life situations, even in individuals that do not develop a MDD or PTSD, we did not find any deployment-related changes in the crucial limbic and prefrontal areas.

In the greater context of PTSD research, the control group is crucial, because it was observed that even trauma-exposed individuals not developing a PTSD may show changes in the processing of negative-affective information in the amygdala [13]. The current study involves a comparison of a trauma-exposed non-PTSD group with a military control group without trauma-exposure in the same time frame. Our findings suggest that if there is a difference in emotional processing between trauma-exposed and non-trauma exposed groups, these differences are more subtle, because we did not observe deployment-related changes in brain activity in our study with a relatively large sample.

Interestingly, Nilsen et al. [33] investigated processing of trauma-specific, negative and neutral stimuli in a group of recent road traffic accident survivors. In line with the results of the current study, no differences between traffic accident survivors and control participants were observed in fronto-limbic areas during processing of trauma-specific compared to negative stimuli (in whole brain and ROI analyses).

### Associations of change scores in limbic neural processing and MDD symptom scores

Even though there were no deployment-related changes in brain activity observed in the main analyses, correlation analyses revealed that only within the combat group the limbic changes in activity patterns were related to changes in MDD symptom scores (and not to PTSD scores). Higher values in the extracted imaging parameter estimates imply that the difference between processing of combat versus negative IAPS stimuli was more similar and less pronounced at posttest compared to pretest. The higher these brain activity changes were, the higher the change scores in depressive symptoms (posttest versus pretest). These results suggest that neural limbic reactivity to trauma-related stimuli in trauma-exposed individuals is related to depressive symptom scores even in this non-clinical sample. Considering the study design in this context, it may be crucial that negative affective stimuli served as control condition. The processing of the negative stimuli also contributed to the results of correlational analyses. Future studies may additionally use neutral stimuli, in order to identify, whether the present results are driven by combat stimuli or the negative affective stimuli. The correlational analysis revealed a relationship between imaging data and behavioral data (depressive symptom score). However, the rating (valence and arousal) of the combat stimuli was not affected by deployment. Thus, future studies may investigate the functional implication of the limbic response in more detail.

In line with previous studies [14–16] with military service members, here we also did not find a relationship between emotion processing in the amygdala and PTSD symptom scores after deployment. To our knowledge, only one study reported such an association [34], however, this was related to processing of positive emotional stimuli (relative to neutral stimuli).

Still, amygdala response was related to MDD symptom scores. A stressful life situation as the deployment to a combat zone may lead to enhanced depressiveness as previously shown in an elevation of depressive symptoms over the course of deployment [28], but no increase in PTSD symptoms can be found. Thus, our results point towards an enhanced relevance of depressive symptoms after deployment of military service members, not only behaviorally, but also on the neural level. Future studies may focus on the development of depression after deployment and changes in neural processing.

### Current study in the context of other studies with soldiers that were deployed to combat regions

In comparison to previous studies with military service members that investigated general emotion processing [14–16, 34], in the current study we investigated the processing of specific combat-related and therewith self-relevant stimuli. In line with these previous studies most soldiers in the present study did not develop PTSD after deployment. In contrast to these studies no deployment-related changes in brain activity between deployed and control group were observed in the limbic-frontal regions. The finding in the correlational analysis implies that changes in processing of combat-related stimuli compared to negative affective stimuli may be more subtle and related to depressive symptoms.

In a previous structural brain imaging study [35] that was conducted with the same sample, volumetric reductions in the ACC, VMPFC and in thalamus were observed in the combat group. In light of the current results, these reductions are not reflected in a change of neural processing of combat-related or negative affective stimuli. Future studies may investigate different functional mechanisms that may be related to these changes in brain structure.

### Strengths and limitations

In the current study we investigated a relatively large sample of military service members before and after deployment as well as a military control group without deployment. We furthermore used standardized stimuli for negative affective processing and previously established combat stimuli.

However, there were also some limitations. First, we were not able to implement a random group assignment. Second, the soldiers prepared for their scheduled deployment independently of study participation. Since the control group also consisted of professional military service members, some participants of the control group were deployed before study participation and may also have been scheduled for deployment in the future. Third, participants may have been concerned about potential implications of questionnaire responses, which may have led to an underreporting of symptoms. Although study staff repeatedly underlined that data is collected for research purposes only, the fear of the stigma of mental illness, which has been shown before [27], may have influenced symptom reporting. Forth, sample sizes between the groups were imbalanced. Fifth, negative affective stimuli served as control condition; however, these stimuli were rated more negative compared to the combat-related stimuli. Therefore, our results possibly over-control for the negative affect.

## Conclusions

The findings of the present study imply that military service members deployment to a war zone is associated with increased symptoms of depression (not PTSD) and that these changes are related to the processing of combat-related stimuli versus negative stimuli in the limbic regions, in particular amygdala and hippocampus. These results highlight the role of depression in individuals that experienced stressful life situations. Future studies may investigate the role of depressiveness after stressful life situations with different tasks that may be more sensitive to changes due to depressive symptoms. Possibly, further investigation of the processing of trauma-related stimulus material and depressive symptoms may be a valuable contribution for a better understanding of the development of psychiatric affective disorders.

In the context of PTSD research our findings are also important, because we neither found significant effects in the group by time interaction nor any correlation between PTSD symptom scores and neural activity. Thus, these findings suggest that there is no difference in emotional processing in limbic and frontal regions between trauma-exposed and non-trauma-exposed individuals.

## Supplementary information

Supplemental Material
