# Supplementary material for: Effects of stress on neural processing of combat-related stimuli in deployed soldiers: an fMRI study
Source: Transl Psychiatry. 2022 Nov 17;12:483. doi: 10.1038/s41398-022-02241-0 (PMC9671957; doi:10.1038/s41398-022-02241-0)
Supplement: Supplementary file 1 — Supplemental Material [file 41398_2022_2241_MOESM1_ESM.docx]

**Supplementary Material**

***Effects of stress on neural processing of combat-related stimuli in deployed soldiers: an fMRI study***

*Robert C. Lorenz^1,2^, Oisin Butler^1^, Gerd Willmund^3^, Ulrich Wesemann^3^, Peter Zimmermann^3^, Jürgen Gallinat^4^, Simone Kühn^1,2,4,5^*

*^1^ Lise Meitner Group for Environmental Neuroscience, Max Planck Institute for Human
Development, Lentzeallee 94, 14195 Berlin, Germany
^2^ Max Planck Dahlem Campus of Cognition (MPDCC), Dillenburgerstr. 53, 14199 Berlin, Germany
^3^ Center for Military Mental Health, Military Hospital Berlin, Scharnhorststr. 13,10115 Berlin,
Germany
^4^ University Medical Center Hamburg-Eppendorf (UKE), Department of Psychiatry and
Psychotherapy W37, Martinistrasse 52, 20246 Hamburg, Germany
^5^ Max Planck-UCL Center for Computational Psychiatry and Ageing Research*

Table of contents

[Methods: Additional questionnaires 2](#_Toc104239430)

[Methods: Examples of experimental stimuli 3](#_Toc104239431)

[Methods: IAPS stimuli 4](#_Toc104239432)

[Results: Tables of results of fMRI analyses 5](#_Toc104239433)

[Results: Task effect 14](#_Toc104239434)

[Results: ROI analysis 15](#_Toc104239435)

[Results: Subgroup analysis 17](#_Toc104239436)

# Methods: Additional questionnaires

*Anxiety Sensitivity Index (ASI; Peterson & Heilbronner, 1987).* The ASI targets the tendency that the experience of anxiety symptoms (somatic and cognitive symptoms) maybe harmful and assess therefore the “fear of fear”. Each of the 16 self-report items is rated on a four point scale ranging from 0 (“very little”) to 4 (“very much”).

*Combat Experience Scale (CES; Hoge et al, 2004; Mental Health Advisory Team, 2006)*. In the current study frequency of different combat experiences is assessed with a version of the CES similar to what is described by the Mental Health Advisory Team (MHAT-IV). In the CES different combat experiences have to be rated in terms of frequency on a five-point scale: “never”, “1”, “2-4”, “5-9”, and “10+”. Experiences are comprised of different stressful combat-related events, e.g.: destroyed housing, working close to mined areas, involvement in combat action including firing at the enemy and coming under fire by the enemy, close combat, seeing dead bodies (or parts of bodies), receiving injuries and seeing wounded women or children.

*Response Styles* *Questionnaire* (*RSQ; Kühner et al., 2007*). The RSQ describes behavioral and cognitive coping styles in response to depressive or dysphoric mood. The questionnaire is based on a theory of response styles that differentiates between rumination and cognitive and behavioral distraction. The long form self-report questionnaire consists of 32 four-point scale items ranging from 1 (“almost never”) to 4 (“almost always”).

*State Trait Anxiety Inventory state (STAI; Spielberger et al., 1970).* The STAI questionnaire assesses trait (general experience) and state (currently experience) anxiety. As the current study is designed to assess changes related to stressful experiences the STAI state anxiety subscale is applied that assesses the temporary state of anxiety and stress. To this end, 20 four-point scale items have to be rated by participant from 1 (“not at all”) to 4 (“very much”).

*References*:

Hoge, C. W., Castro, C. A., Messer, S. C., McGurk, D., Cotting, D. I., & Koffman, R. L. (2004). Combat duty in Iraq and Afghanistan, mental health problems, and barriers to care. *New England journal of medicine*, *351*(1), 13-22.

Kühner, C., Huffziger, S., & Nolen-Hoeksema, S. (2007). *Response styles questionnaire: RSQ-D*. Hogrefe.

Mental Health Advisory Team (2006). Mental Health Advisory Team (MHAT)-IV Operation Iraqi Freedom, 05–07. *Washington, DC: US Army Medical Command*.

Peterson, R. A., & Heilbronner, R. L. (1987). The anxiety sensitivity index: Construct validity and factor analytic structure. *Journal of anxiety disorders*, *1*(2), 117-121.

Spielberger, C. D., Gorsuch, R., & Lushene, R. (1970). The State-Trait Anxiety Inventory (STAI) test manual. Palo Alto.

# Methods: Examples of experimental stimuli


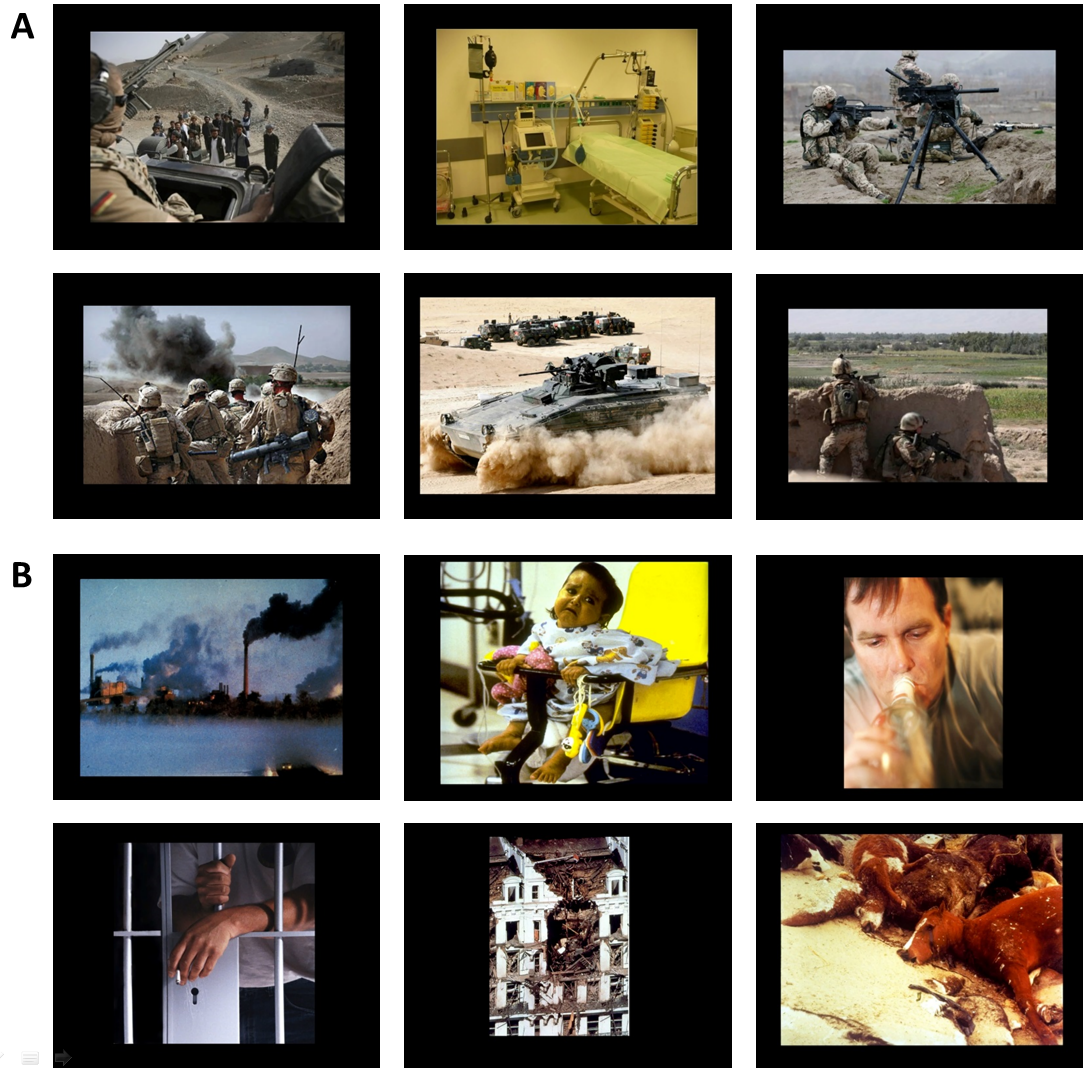


Figure S1: Examples of experimental stimuli for combat-related stimuli (A) and negative affect-related IAPS stimuli (B).

# Methods: IAPS stimuli

For the IAPS stimulus set the following IAPS pictures with their number were taken:

- Toddler: 2095
- Drug addict: 2710
- Eye disease: 3160
- Burn victim: 3215
- Dying man: 3230
- Infant: 3350
- Aimed gun: 6210
- Knife: 6300
- Suicide: 6570
- Dead cows: 9181
- Dirty: 9300
- Assault: 9429
- Ruins: 9470
- Duck in oil: 9560
- Fire: 9925
- Mother: 2312
- Sad Girls: 2455
- Man: 2490
- Elderly Woman: 2590
- Refugees: 2695
- Jail: 2722
- Alcoholic: 2753
- Disabled child: 3300
- Jail: 6010
- Barbed wire: 9010
- Wires: 9080
- Smoke: 9280
- Garbage: 9290
- Homeless Man: 9331
- Empty Pool: 9360

# Results: Tables of results of fMRI analyses

Table S1: Task effect (pretest) for processing of combat-related stimuli across all participants; significance threshold p (FWE corrected) < 0.05.

| Brain structure (peak) | Hem | Cluster size | T (peak) | p (FWE) | X | Y | Z |
| --- | --- | --- | --- | --- | --- | --- | --- |
| Occipital fusiform gyrus | R/L | 19633* | 189.65 | <0.001 | 24 | -76 | -10 |
| Temporal occipital fusiform cortex |  |  | 187.04 | <0.001 | 30 | -61 | -10 |
| Temporal occipital fusiform cortex |  |  | 186.88 | <0.001 | -27 | -61 | -10 |
| Cerebellum | L | 7 | 11.23 | <0.001 | -18 | -37 | -40 |
| Postcentral Gyrus | R | 2 | 7.98 | <0.001 | 39 | -25 | 65 |
| Postcentral Gyrus | L | 1 | 6.15 | <0.001 | -63 | -7 | 17 |
| Frontal Pole | R | 2 | 5.64 | 0.002 | 27 | 38 | 47 |

* Cluster extents into visual and dorsal stream, precuneus, parietal areas (superior parietal cortex, inferior parietal cortex), temporal areas (middle and superior temporal gyrus), frontal areas (supplementary motor cortex, cingulate cortex, medial PFC, VMPFC, IFG extending into insular cortex, precentral gyrus, and superior frontal gyrus), and subcortical areas (thalamus, caudate, amygdala, and hippocampus).

Table S2: Task effect (pretest) for processing of negative affective stimuli across all participants; significance threshold p (FWE corrected) < 0.05.

| Brain structure (peak) | Hem | Cluster size | T (peak) | p (FWE) | X | Y | Z |
| --- | --- | --- | --- | --- | --- | --- | --- |
| Occipital fusiform gyrus | R/L | 18229* | 194.23 | <0.001 | 24 | -76 | -10 |
| Temporal occipital fusiform cortex |  |  | 181.15 | <0.001 | 30 | -61 | -10 |
| Occipital fusiform gyrus |  |  | 180.16 | <0.001 | -27 | -76 | -10 |
| Frontal medial cortex | R | 173 | 34.45 | <0.001 | 3 | 44 | -16 |
| Frontal superior medial cortex | L | 464 | 22.36 | <0.001 | -12 | 56 | 17 |
|  |  |  | 21.15 | <0.001 | -6 | 53 | 29 |
|  |  |  | 20.84 | <0.001 | -9 | 53 | 38 |
| Cerebellum | L | 7 | 11.82 | <0.001 | -18 | -37 | -40 |
| Thalamus | R | 2 | 7.51 | <0.001 | 9 | -10 | 17 |
| Posterior cingulate gyrus | R | 4 | 6.19 | <0.001 | 6 | -37 | 32 |
| Postcentral Gyrus | R | 1 | 6.15 | <0.001 | 39 | -25 | 65 |
| Thalamus | L | 1 | 6.07 | <0.001 | -6 | -13 | 17 |
| Superior frontal gyrus | L | 1 | 5.89 | <0.001 | -12 | 26 | 62 |
| Caudate Nucleus | R | 1 | 4.95 | 0.049 | 9 | 23 | 5 |

* Cluster extents into visual and dorsal stream, precuneus, parietal areas (superior parietal cortex, inferior parietal cortex), temporal areas (middle and superior temporal gyrus), frontal areas (supplementary motor cortex, cingulate cortex, medial PFC, VMPFC, IFG extending into insular cortex, precentral gyrus, and superior frontal gyrus), and subcortical areas (thalamus, caudate, amygdala, and hippocampus).

Table S3: Task effect (pretest) for processing of combat-related stimuli vs. negative affective stimuli across all participants; significance threshold p (FWE corrected) < 0.05.

| Brain structure (peak) | Hem | Cluster size | T (peak) | p (FWE) | X | Y | Z |
| --- | --- | --- | --- | --- | --- | --- | --- |
| Precuneus | R/L | 5858 | 22.45 | <0.001 | 18 | -55 | 14 |
| Lateral occipital cortex |  |  | 22.32 | <0.001 | 42 | -73 | 29 |
| Precuneus |  |  | 21.71 | <0.001 | -15 | -58 | 14 |
| Superior frontal gyrus | R | 369 | 15.66 | <0.001 | 24 | 26 | 44 |
| Superior frontal gyrus |  |  | 8.90 | <0.001 | 27 | 17 | 59 |
| Ventromedial prefrontal cortex | R/L | 183 | 9.60 | <0.001 | 3 | 50 | -10 |
| Subcallosal cortex |  |  | 7.13 | <0.001 | -9 | 29 | -13 |
| Ventromedial prefrontal cortex |  |  | 6.41 | <0.001 | -12 | 41 | -10 |
| Anterior middle temporal gyrus | R | 95 | 9.51 | <0.001 | 57 | -4 | -19 |
| Superior frontal gyrus | L | 112 | 9.33 | <0.001 | -24 | 29 | 44 |
|  |  |  | 7.16 | <0.001 | -21 | 17 | 44 |
| Middle temporal gyrus, temporo-occipital part | L | 42 | 8.61 | <0.001 | -54 | -55 | -7 |
| Middle frontal gyrus | R | 20 | 6.14 | <0.001 | 36 | 11 | 29 |
| Subcallosal cortex | R/L | 3 | 5.99 | <0.001 | 0 | 17 | -4 |
| Posterior temporal fusiform cortex | R | 4 | 5.97 | <0.001 | 42 | -13 | -28 |
| Thalamus | R | 3 | 5.82 | 0.001 | 18 | -31 | 5 |
| Posterior temporal fusiform cortex | L | 6 | 5.50 | 0.004 | -57 | -10 | -22 |
| Frontal pole | R | 2 | 5.46 | 0.005 | 24 | 50 | -1 |
| Caudate nucleus | R | 2 | 5.45 | 0.005 | 15 | 29 | 2 |
| Intracalcarine cortex | R | 2 | 5.36 | 0.008 | 15 | -88 | 5 |
| Postcentral Gyrus | L | 1 | 5.02 | 0.037 | -9 | -37 | 74 |

Table S4: Task effect (pretest) for processing of negative affective stimuli vs. combat-related stimuli across all participants; significance threshold p (FWE corrected) < 0.05.

| Brain structure (peak) | Hem | Cluster size | T (peak) | p (FWE) | X | Y | Z |
| --- | --- | --- | --- | --- | --- | --- | --- |
| Lingual gyrus | R/L | 509 | 13.91 | <0.001 | 3 | -76 | -1 |
|  |  |  | 7.16 | <0.001 | -18 | -76 | -7 |
| Occipital fusiform gyrus |  |  | 6.27 | <0.001 | 21 | -73 | -13 |
| Hippocampus | L | 2306 | 13.45 | <0.001 | -18 | -7 | -16 |
| Frontal orbital cortex |  |  | 11.84 | <0.001 | -42 | 26 | -16 |
|  |  |  | 11.51 | <0.001 | -48 | 26 | -7 |
| Parahippocampal gyrus | R | 680 | 11.43 | <0.001 | 21 | -4 | -16 |
| Putamen |  |  | 9.59 | <0.001 | 15 | 8 | -7 |
| Inferior frontal gyrus |  |  | 9.51 | <0.001 | 51 | 29 | -7 |
| Cerebellum | R | 47 | 8.03 | <0.001 | 27 | -70 | -31 |
|  |  |  | 6.48 | <0.001 | 12 | -76 | -28 |
| Anterior supramarginal gyrus | R | 96 | 7.78 | <0.001 | 63 | -19 | 29 |
|  |  |  | 7.14 | <0.001 | 57 | -22 | 47 |
|  |  |  | 6.47 | <0.001 | 54 | -22 | 35 |
| Middle temporal gyrus, posterior division | R | 37 | 7.77 | <0.001 | 48 | -34 | -1 |
| inferior lateral occipital cortex | R | 34 | 7.17 | <0.001 | 39 | -76 | -13 |
| Middle temporal gyrus, posterior division | L | 9 | 5.87 | 0.001 | -51 | -28 | -7 |
| Postcentral gyrus | L | 7 | 5.81 | 0.001 | -57 | -22 | 29 |
| Middle temporal gyrus, posterior division | L | 4 | 5.60 | 0.003 | -54 | -40 | -1 |
| Corpus callosum | R | 2 | 5.57 | 0.003 | 9 | -31 | 17 |
| Thalamus | L | 2 | 5.50 | 0.004 | -6 | -22 | -1 |
| Thalamus | L | 2 | 5.28 | 0.012 | -6 | -16 | 11 |
| Middle cingulate gyrus | L | 2 | 5.25 | 0.013 | -3 | -16 | 35 |
| Thalamus | L | 2 | 5.19 | 0.017 | -3 | -16 | 2 |
| Occipital fusiform gyrus | L | 2 | 5.15 | 0.021 | -36 | -73 | -13 |
| Thalamus | L | 1 | 5.14 | 0.022 | -3 | -25 | 2 |
| Thalamus | L | 1 | 5.13 | 0.022 | -9 | -13 | 5 |
| Temporal pole | L | 4 | 5.13 | 0.022 | -48 | 8 | -28 |
| Middle frontal gyrus | L | 1 | 5.11 | 0.025 | -42 | 26 | 32 |
| inferior lateral occipital cortex | L | 2 | 5.05 | 0.032 | -36 | -82 | -10 |
| Temporal pole | R | 1 | 5.04 | 0.034 | 36 | 8 | -22 |

Table S5: Combat group posttest of processing of combat-related stimuli vs. negative affective stimuli; significance threshold p (FWE corrected) < 0.05.

| Brain structure (peak) | Hem | Cluster size | T (peak) | p (FWE) | X | Y | Z |
| --- | --- | --- | --- | --- | --- | --- | --- |
| Precuneus | R/L | 6874 | 47.56 | <0.001 | -15 | -58 | 17 |
|  |  |  | 46.54 | <0.001 | 15 | -55 | 17 |
|  |  |  | 40.19 | <0.001 | 6 | -61 | 23 |
| Superior frontal gyrus | R | 387 | 20.49 | <0.001 | 24 | 26 | 44 |
| Ventromedial prefrontal cortex | R/L | 270 | 20.26 | <0.001 | 6 | 47 | -10 |
|  |  |  | 12.92 | <0.001 | -9 | 47 | -10 |
| Superior frontal gyrus | L | 174 | 13.32 | <0.001 | -21 | 29 | 41 |
|  |  |  | 7.68 | <0.001 | -24 | 17 | 53 |
| Frontal pole | R | 36 | 8.95 | <0.001 | 24 | 53 | 5 |
| Middle temporal gyrus, posterior division | L | 31 | 7.81 | <0.001 | -51 | -13 | -19 |
| Precentral gyrus | R | 14 | 6.64 | <0.001 | 36 | 8 | 29 |
| Intracalacarine Cortex | R | 9 | 6.41 | <0.001 | 15 | -85 | 2 |
| Caudate Nucleus | R | 4 | 6.14 | <0.001 | 9 | 23 | 5 |
| Subcallosal Cortex | L | 1 | 5.85 | 0.001 | -3 | 11 | -7 |
| Thalamus | R | 1 | 5.83 | 0.001 | 18 | -31 | 5 |
| Precentral gyrus | R | 4 | 5.63 | 0.002 | 27 | -22 | 65 |
| Precentral gyrus | R | 3 | 5.56 | 0.003 | 3 | -25 | 68 |
| Subcallosal Cortex | R/L | 3 | 5.42 | 0.006 | 0 | 14 | -4 |
| Precentral gyrus | R | 5 | 5.34 | 0.009 | 15 | -25 | 65 |
| Subcallosal Cortex | R | 1 | 5.09 | 0.028 | 6 | 20 | -7 |

Table S6: Combat group posttest of processing of negative affective stimuli vs. combat-related stimuli stimuli; significance threshold p (FWE corrected) < 0.05.

| Brain structure (peak) | Hem | Cluster size | T (peak) | p (FWE) | X | Y | Z |
| --- | --- | --- | --- | --- | --- | --- | --- |
| Lingual gyrus | R/L | 1299 | 24.03 | <0.001 | 3 | -79 | -1 |
|  |  |  | 18.41 | <0.001 | -3 | -70 | -1 |
| Supracalcarine cortex |  |  | 17.4 | <0.001 | 3 | -88 | 14 |
| Frontal orbital cortex | L | 6032 | 21.35 | <0.001 | -42 | 26 | -16 |
|  |  |  | 19.69 | <0.001 | -42 | 26 | -4 |
|  |  |  | 16.67 | <0.001 | -33 | 20 | -19 |
| Middle temporal gyrus, posterior division | R | 204 | 18.04 | <0.001 | 51 | -31 | -4 |
| Middle temporal gyrus, temporo-occipital part |  |  | 15.77 | <0.001 | 45 | -37 | 2 |
| Postecentral gyrus | R | 447 | 14.26 | <0.001 | 63 | -19 | 26 |
| Anterior supramarginal gyrus |  |  | 11.57 | <0.001 | 60 | -25 | 44 |
|  |  |  | 11.5 | <0.001 | 54 | -31 | 50 |
| Middle temporal gyrus, posterior division | L | 190 | 12.76 | <0.001 | -48 | -31 | -4 |
|  |  |  | 9.63 | <0.001 | -57 | -25 | -4 |
| Inferior temporal gyrus, posterior division | L | 545 | 10.89 | <0.001 | -57 | -25 | 29 |
| Posterior supramarginal gyrus |  |  | 10.28 | <0.001 | -54 | -46 | 47 |
|  |  |  | 9.6 | <0.001 | -60 | -43 | 29 |
| Cerebellum | L | 68 | 7.72 | <0.001 | -24 | -73 | -28 |
|  |  |  | 6.54 | <0.001 | -18 | -79 | -28 |
|  |  |  | 6.1 | <0.001 | -36 | -73 | -19 |
| Cerebellum | L | 64 | 7.62 | <0.001 | -42 | -58 | -31 |
|  |  |  | 7.19 | <0.001 | -33 | -55 | -31 |
| Middle cingulate gyrus | L | 15 | 7.5 | <0.001 | -3 | -16 | 38 |
| Precentral gyrus | R | 19 | 7.45 | <0.001 | 54 | 2 | 41 |
|  |  |  | 5.54 | <0.001 | 48 | -1 | 50 |
| Inferior lateral occipital cortex | L | 19 | 7.26 | <0.001 | -39 | -85 | -10 |
| Corpus callosum | R | 15 | 6.81 | <0.001 | 18 | -37 | 17 |
| Precentral gyrus | R | 8 | 6.43 | <0.001 | 30 | -10 | 50 |
| Middle frontal gyrus | R | 3 | 5.76 | 0.001 | 39 | 23 | 38 |
| Brainstem | L | 3 | 5.66 | 0.002 | -3 | -22 | -34 |
| Heschl’s gyrus | R | 2 | 5.44 | 0.006 | 51 | -13 | 2 |
| Corpus callosum | L | 4 | 5.43 | 0.006 | -15 | -40 | 17 |
| Middle temporal gyrus, anterior division | L | 1 | 5.37 | 0.008 | -48 | -4 | -31 |
| Thalamus | R | 1 | 5.24 | 0.014 | 6 | -28 | 2 |
| Angular gyrus | R | 1 | 5.19 | 0.018 | 57 | -52 | 41 |
| Precuneus | L | 1 | 5.09 | 0.027 | -21 | -46 | 14 |
| Posterior cingulate gyrus | R/L | 1 | 5.04 | 0.033 | 0 | -31 | -40 |
| White matter | L | 1 | 4.98 | 0.044 | -30 | -25 | 5 |
| Insular cortex | R | 1 | 4.97 | 0.046 | 39 | -7 | 5 |

Table S7: Control group posttest of processing of combat-related stimuli vs. negative affective stimuli; significance threshold p (FWE corrected) < 0.05.

| Brain structure (peak) | Hem | Cluster size | T (peak) | p (FWE) | X | Y | Z |
| --- | --- | --- | --- | --- | --- | --- | --- |
| Precuneus | R/L | 4089 | 23.04 | <0.001 | -15 | -61 | 17 |
|  |  |  | 22.81 | <0.001 | 18 | -55 | 17 |
| Superior lateral occipital cortex |  |  | 20.16 | <0.001 | -39 | -79 | 29 |
| Posterior parahippocampal gyrus | R | 234 | 19.31 | <0.001 | 27 | -37 | -13 |
| Anterior parahippocampal gyrus |  |  | 6.8 | <0.001 | 24 | -22 | -22 |
| Middle frontal gyrus | R | 310 | 14.03 | <0.001 | 27 | 29 | 44 |
| Superior frontal gyrus |  |  | 9.1 | <0.001 | 27 | 11 | 56 |
|  |  |  | 5.83 | 0.001 | 24 | 8 | 65 |
| Superior frontal gyrus | L | 63 | 10.3 | <0.001 | -24 | 26 | 38 |
| Middle temporal gyrus, anterior division | R | 58 | 9.13 | <0.001 | 54 | -4 | -19 |
| Ventromedial prefrontal cortex | R/L | 78 | 7.92 | <0.001 | 6 | 53 | -7 |
|  |  |  | 6.96 | <0.001 | -6 | 56 | -4 |
|  |  |  | 6.76 | <0.001 | 9 | 41 | -13 |
| Superior frontal cortex | L | 15 | 7.58 | <0.001 | -18 | -10 | 71 |
| Frontal pole | R | 24 | 7.37 | <0.001 | 27 | 53 | 2 |
| Middle temporal gyrus, temporo-occipital part | L | 5 | 6.3 | <0.001 | -57 | -52 | -7 |
|  |  |  | 6 | <0.001 | -57 | -61 | -4 |
| Middle temporal gyrus, anterior division | L | 7 | 6.06 | <0.001 | -60 | -10 | -19 |
| Middle temporal gyrus, posterior division | R | 1 | 5.75 | 0.001 | 60 | -37 | -10 |
| Anterior parahippocampal gyrus | L | 7 | 5.72 | 0.002 | -18 | -19 | -22 |
| Middle frontal gyrus | R | 2 | 5.51 | 0.004 | 36 | 2 | 50 |
| Precentral gyrus | L | 4 | 5.49 | 0.004 | -36 | -10 | 62 |
| White matter | L | 1 | 5.32 | 0.01 | -18 | 35 | -1 |
| Subcallosal cortex | RL | 1 | 5.16 | 0.019 | 0 | 14 | -4 |
| Middle temporal gyrus, posterior division | R | 1 | 5.04 | 0.034 | 57 | -34 | -13 |
| Intracalcarine cortex | R | 1 | 4.96 | 0.048 | 15 | -85 | 2 |

Table S8: Control group posttest of processing of negative affective stimuli vs. combat-related stimuli stimuli; significance threshold p (FWE corrected) < 0.05.

| Brain structure (peak) | Hem | Cluster size | T (peak) | p (FWE) | X | Y | Z |
| --- | --- | --- | --- | --- | --- | --- | --- |
| Hippocampus | L | 998 | 13.08 | <0.001 | -18 | -7 | -16 |
| Frontal orbital cortex |  |  | 11.59 | <0.001 | -45 | 32 | -13 |
|  |  |  | 11.31 | <0.001 | -45 | 23 | -13 |
| Lingual gyrus | R/L | 607 | 13.06 | <0.001 | 3 | -79 | -1 |
| Occipital pole |  |  | 12.69 | <0.001 | -6 | -94 | 23 |
|  |  |  | 11.59 | <0.001 | -3 | -91 | 11 |
| Inferior lateral occipital cortex | R | 77 | 10.29 | <0.001 | 45 | -70 | -13 |
|  |  |  | 7.67 | <0.001 | 39 | -79 | -7 |
| Temporal Occipital Fusiform Cortex |  |  | 6.1 | <0.001 | 45 | -55 | -19 |
| Frontal orbital cortex | R | 745 | 10.23 | <0.001 | 33 | 23 | -19 |
| Temporal pole |  |  | 9.64 | <0.001 | 36 | 5 | -19 |
|  |  |  | 8.9 | <0.001 | 51 | 14 | -7 |
| Anterior cingulate cortex | L | 898 | 10.13 | <0.001 | -3 | 26 | 26 |
| Superior frontal gyrus |  |  | 9.57 | <0.001 | -6 | 56 | 17 |
|  |  |  | 9.36 | <0.001 | -6 | 56 | 29 |
| Superior temporal gyrus, posterior division | R | 62 | 10.05 | <0.001 | 51 | -31 | -1 |
| Superior temporal gyrus, posterior division | L | 49 | 7.4 | <0.001 | -54 | -31 | -1 |
|  |  |  | 6.55 | <0.001 | -51 | -40 | -4 |
| Occipital fusiform gyrus | L | 36 | 7.39 | <0.001 | -33 | -70 | -16 |
|  |  |  | 6.04 | <0.001 | -33 | -82 | -13 |
| Posterior supramarginal gyrus | L | 10 | 7.38 | <0.001 | -57 | -49 | 38 |
| Anterior supramarginal gyrus | R | 4 | 6.4 | <0.001 | 54 | -31 | 50 |
| Middle cingulate gyrus | R/L | 9 | 6.37 | <0.001 | 0 | -16 | 35 |
| Temporal pole | L | 19 | 6.36 | <0.001 | 48 | 20 | -28 |
|  |  |  | 5.73 | 0.001 | 48 | 11 | -25 |
| Cerebellum | L | 8 | 6.33 | <0.001 | -42 | -58 | -31 |
| Thalamus | R | 16 | 6.16 | <0.001 | 3 | -19 | 5 |
| Caudate Nucleus | L | 6 | 6.07 | <0.001 | 15 | 17 | 8 |
| Temporal fusiform cortex, posterior division | R | 2 | 6.03 | <0.001 | -39 | -40 | -19 |
| Insular cortex | R | 7 | 6.02 | <0.001 | 30 | -22 | 5 |
| Postcentral gyrus | R | 4 | 5.72 | 0.001 | 63 | -13 | 17 |
| Cerebellum | R | 7 | 5.69 | 0.002 | 15 | -76 | -31 |
| Caudate Nucleus | R | 5 | 5.69 | 0.002 | 15 | -1 | 17 |
| Postcentral gyrus | R | 8 | 5.68 | 0.002 | 63 | -19 | 26 |
| Insular cortex | R | 6 | 5.6 | 0.003 | 33 | 2 | 11 |
| Thalamus | R | 4 | 5.54 | 0.004 | 21 | -16 | 17 |
| Postcentral gyrus | L | 3 | 5.49 | 0.004 | -54 | -19 | 32 |
| Cerebellum | R | 6 | 5.45 | 0.005 | 21 | -70 | -31 |
| Precentral gyrus | L | 4 | 5.38 | 0.007 | -48 | 5 | 29 |
| Insular cortex | L | 3 | 5.27 | 0.012 | -39 | -10 | -4 |
| Insular cortex | L | 3 | 5.26 | 0.013 | -30 | -22 | -1 |
| Precentral gyrus | R | 1 | 5.23 | 0.014 | 51 | -1 | 47 |
| Occipital fusiform gyrus | R | 2 | 5.18 | 0.018 | 18 | -82 | -10 |
| Hippocampus | R | 1 | 5.17 | 0.019 | 30 | -13 | -7 |
| Insular cortex | R | 1 | 5.14 | 0.021 | 39 | -7 | -10 |
| Occipital fusiform gyrus | R | 1 | 5.11 | 0.024 | 33 | -67 | -22 |
| Anterior supramarginal gyrus | R | 1 | 5.09 | 0.027 | 60 | -28 | 47 |
| Cerebellum | R | 1 | 4.98 | 0.043 | 6 | -49 | -31 |

# Results: Task effect

**
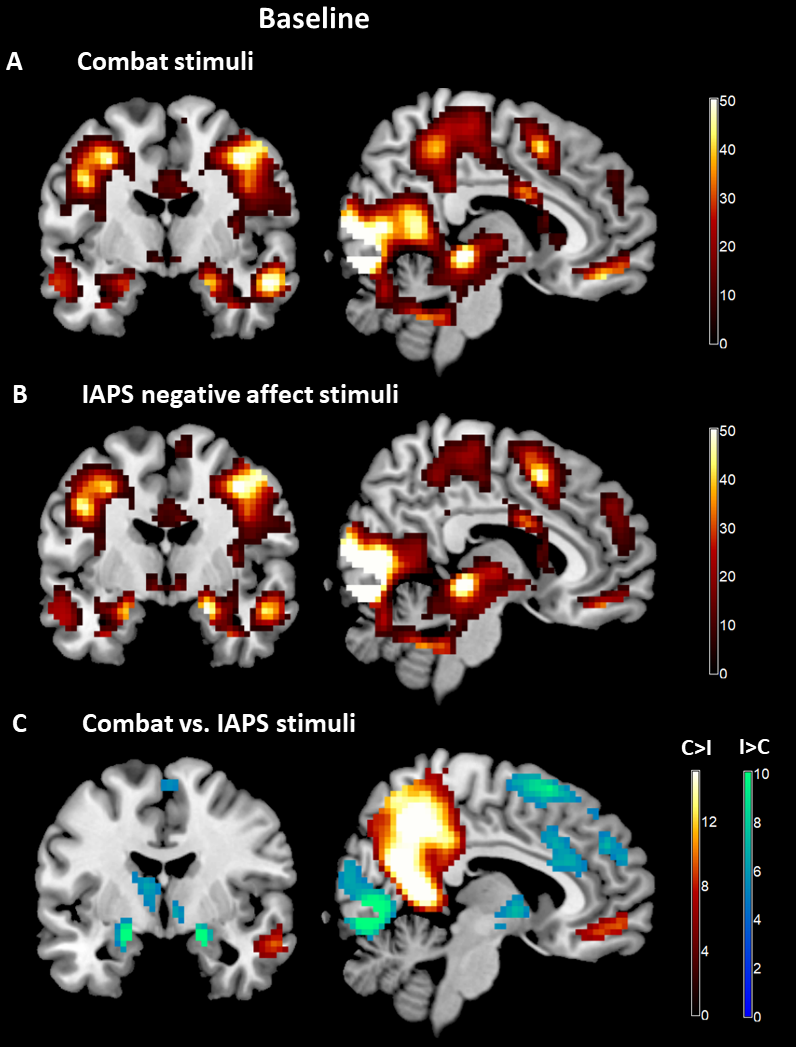
**

Figure S2: fMRI task effect at baseline across all participants. (A) Processing of combat and (B) negative affective IAPS were accompanied by activity in a large cortical network involving midline structures such as posterior parietal cortex, supplementary motor cortex, medial prefrontal cortex and VMPFC. (C) The differential effect shows stronger activity in precuneus posterior parietal cortex, and VMPFC at presentation of combat stimuli (versus IAPS) as well as stronger activity in supplementary motor cortex, anterior cingulate gyrus, medial prefrontal cortex, and amygdala at presentation of IAPS stimuli (versus combat).

# Results: ROI analysis


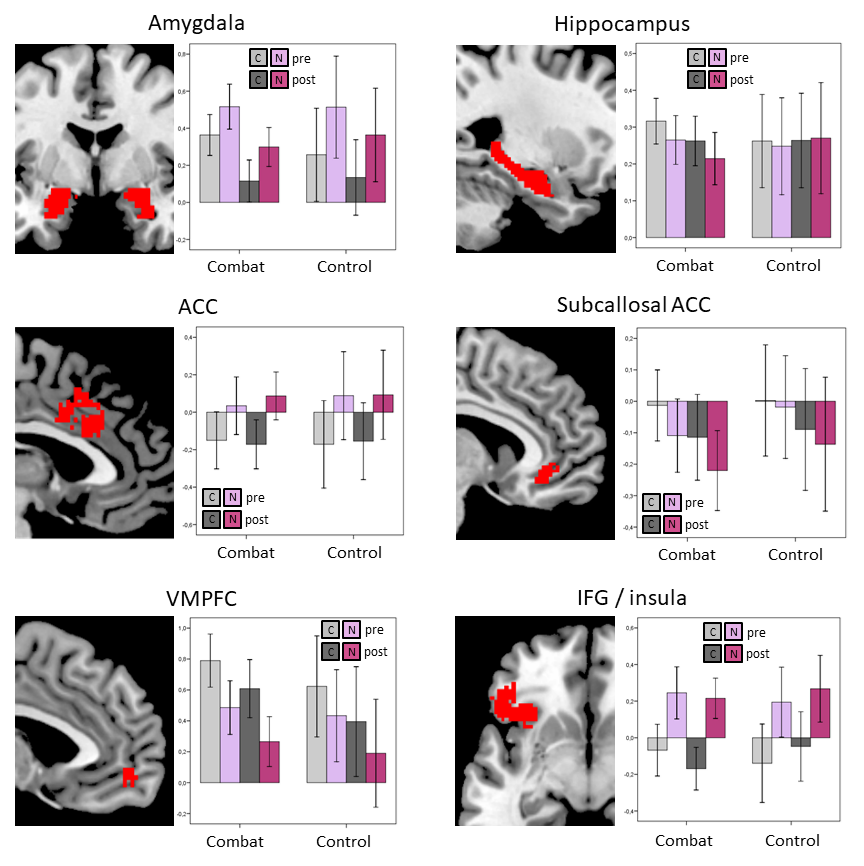


Figure S3: Imaging results, region of interest (ROI) analysis. Anatomical location and bar graphs for the contrast combat versus negative affective IAPS stimuli for the two groups and time points were presented for the a priori defined ROIs amygdala, hippocampus, anterior cingulate cortex (ACC), subcallsol ACC, ventromedial prefrontal cortex (VMPFC) and inferior frontal gyrus (IFG) / insula (left). In all presented ROI analyses, no significant group (combat vs. control group) by time (pretest versus posttest) interactions were observed. Error bars represent 95% confidence interval. Grey bars represent combat stimuli (“C”; light grey: pretest, dark grey: posttest) and pink bars represent negative affective stimuli (“N”; light pink: pretest, dark pink: posttest).

Table S9: Imaging results, region of interest (ROI) analysis of group x stimulus type x time resulting from a repeated measures analysis of variance.

| **Anatomical region** | **ME group (F; p)** | **ME time (F; p)** | **ME stimulus type (F; p)** | **Interaction time x group (F; p)** | **Interaction stimulus type x group (F; p)** | **Interaction time x stimulus type (F; p)** | **Three-way interaction (F; p)** |
| --- | --- | --- | --- | --- | --- | --- | --- |
| **Frontal regions** | | | | |  |  |  |
| ACC | 0.016; 0.899 | 0.029; 0.866 | 86.631; <0.001 | 0.001; .974 | 0.401; 0.527 | 0.407; 0.524 | 0.813; 0.369 |
| Subcallosal ACC | 0.355; 0.552 | 2.123; 0.147 | 9.724; 0.002 | <0.001; 0.994 | 2.402; 0.124 | 0.257; 0.613 | 0.053; 0.818 |
| VMPFC | 0.739; 0.391 | 6.054; 0.015 | 52.110; <0.001 | 0.038; 0.845 | 3.064; 0.082 | 0.329; 0.567 | 0.060; 0.807 |
| Inferior frontal cortex / insula left | 0.017; 0.897 | 0.015; 0.902 | 215.940; <0.001 | 1.159; 0.284 | 0.276; 0.6 | 0.458; 0.5 | 1.350; 0.247 |
| Inferior frontal cortex / insula right | 0.151; 0.698 | <0.000; 0.985 | 131.789; <0.001 | 0.258; 0.613 | <0.000; 0.99 | 0.813; 0.369 | 0.613; 0.435 |
| **Subcortical regions** | | | | |  |  |  |
| Amygdala left | 0.239; 0.626 | 6.408; 0.012 | 139.588; <0.001 | 0.242; 0.624 | 5.081; 0.026 | 0.039; 0.843 | 0.901; 0.344 |
| Amygdala right | 0.003; 0.956 | 6.033; 0.015 | 125.076; <0.001 | 0.144; 0.705 | 2.595; 0.11 | 0.052; 0.819 | 0.119; 0.731 |
| Hippocampus left | 0.108; 0.742 | 0.336; 0.563 | 0.090; 0.765 | 3.11; 0.08 | 7.954; 0.006 | 0.8; 0.373 | 0.605; 0.438 |
| Hippocampus right | 0.005; 0.946 | 0.209; 0.648 | 4.255; 0.041 | 0.527; 0.469 | 3.141; 0.079 | 0.242; 0.623 | 0.121; 0.728 |

As shown in supplemental Figure S3 and Table S9 a decrease over time was observed across the groups in amygdala and VMPFC. In both sessions the same stimulus set was used, therefore, possible effects like stimulus novelty may be related to this decrease. In line with this interpretation, a previous study showed that activity the amygdala is modulated by stimulus novelty, in particular negative affective novel stimuli.^[[1]](#footnote-1)^

# Results: Subgroup analysis

*Since all participants in the combat group were deployed in a crisis area, they were exposed to a high risk for the experience of a potential traumatic event. As described in the methods section of the manuscript, the Combat Experience Scale was assessed. In this self-report questionnaire participants were instructed to rate the frequency of different combat experiences on a five-point scale (“never”, “1”, “2-4”, “5-9”, and “10+”). We used this scale in order to divide the combat group into different types of combat experience and computed different subscales that were previously identified in a principle component analysis in a sample of 191 deployed soldiers (Hellenthal et al., 2017)^^[[2]](#footnote-2)^^. In this study the following four subscales/factors were identified:*

- *Factor 1: Combat, fighting*
- *Factor 2: Threatening environment in operation*
- *Factor 3: Confrontation with hardship, suffering, violence in the population*
- *Factor 4: Dealing with serious injuries, death*

*Experiences in factor 1 and 2 are more related to direct combat involvement, while the other two factors (3 and 4) are more related to witnessing consequences of war to civilians and wartime comrades. We clustered the deployed soldiers into four groups: Group “both” with at least one experience in factors 1 and 2 as well as in factors 3 and 4 (n=54), group “only direct combat” with at least one experience in factors 1 and 2 and no experience in factors 3 and 4 (n=0), group “only consequences of war” with at least one experience in factors 3 and 4 and no experience in factors 1 and 2 (n=40), and group “no experience” that participants reported no experience in any items of the four factors (n=10). When calculating analyses of variance with repeated measures and the factors group (between-subject) and time (within-subject) with the differential contrast images (combat vs. negative affective stimuli) for each of the ROIs, no significant group by time interactions were observed (see table R1).*

**Table R1: Results of subgroup analysis with the ROIs.**

| **Anatomical region** | **Interaction: F; p** |
| --- | --- |
| **Frontal regions** |  |
| ACC | 1,872; 0,137 |
| Subcallosal ACC | 0,204; 0,894 |
| VMPFC | 0,189; 0,904 |
| Inferior frontal cortex / insula left | 1,495; 0,219 |
| Inferior frontal cortex / insula right | 0,755; 0,521 |
| **Subcortical regions** |  |
| Amygdala left | 0,307; 0,820 |
| Amygdala right | 0,150; 0,930 |
| Hippocampus left | 1,453; 0,230 |
| Hippocampus right | 0,854; 0,467 |

1. Weierich, M. R., Wright, C. I., Negreira, A., Dickerson, B. C., & Barrett, L. F. (2010). Novelty as a dimension in the affective brain. *Neuroimage*, *49*(3), 2871-2878. [↑](#footnote-ref-1)
2. Hellenthal, A., Zimmermann, P., Willmund, G., Lovinusz, A., Fiebig, R., Bozoyan, C., Maercker, A., & Alliger-Horn, C. (2017). Einsatzerlebnisse, moralische Verletzungen, Werte und psychische Erkrankungen bei Einsatzsoldaten der Bundeswehr. Verhaltenstherapie, 27(4), 244–252. https://doi.org/10.1159/000470848 [↑](#footnote-ref-2)
